# Association Between State‐Wide Cardiac Quality Improvement Program and Costs Following Intervention for Coronary Artery Disease

**DOI:** 10.1002/clc.70030

**Published:** 2024-11-18

**Authors:** Edwin S. Wong, Joshua Nelson, Richard Whitten, Charles Maynard, Jeannie Collins‐Brandon, Kristin Sitcov, Ravi S. Hira

**Affiliations:** ^1^ Department of Health Systems and Population Health University of Washington Seattle Washington USA; ^2^ Noridian Healthcare Solutions, LLC Fargo North Dakota USA; ^3^ Foundation for Health Care Quality Seattle Washington USA; ^4^ Pulse Heart Institute and Multicare Health System Tacoma Washington USA

**Keywords:** coronary artery disease, costs, economic analysis, Medicare, percutaneous coronary intervention

## Abstract

**Background:**

Since 2010, all non‐VA hospitals performing cardiac surgeries and percutaneous interventions in Washington State have participated in the Cardiac Care Outcomes Assessment Program (COAP), a data‐driven, physician‐led collaborative quality improvement (QI) collaborative. Prior literature has demonstrated QI programs such as COAP can avert avoidable utilization such as hospital readmissions. However, it is unknown whether such improvements translate into economic benefits.

**Hypothesis:**

This study compared downstream healthcare costs between patients undergoing cardiac interventions for coronary artery disease (CAD) at hospitals that were and were not participating in COAP.

**Methods:**

Post hoc analysis of Medicare administrative and claims data examined 2.5 million randomly selected deidentified beneficiaries receiving a percutaneous coronary intervention or coronary artery bypass grafting between 2013 and 2020. Total costs were defined as all reimbursements paid by Medicare for up to 5 years following cardiac intervention. Because all non‐VA hospitals in Washington State participated in COAP, we compared respective groups of patients receiving intervention in Washington State with all non‐Washington states, adjusting for patient demographics and comorbidity. To model costs, we applied a multipart estimator, which distinguishes the impact of QI program participation due to survival and utilization while accounting for censoring.

**Results:**

Total 5‐year downstream costs were $3861 lower (95% confidence interval [CI] = $1794 to $5741) among patients receiving cardiac intervention at COAP‐exposed hospitals. Lower costs were largely driven by lower utilization during calendar quarters where death was not observed.

**Conclusions:**

Participation in this state‐wide cardiac quality improvement program was associated with economic benefits in patients receiving intervention for CAD.

## Graphical abstract

In this observational study, we examined whether hospital participation in cardiac quality improvement collaboratives was associated with reductions in medical costs among patients receiving CABG or PCI. Using econometric methods, we found patients receiving intervention from participating hospitals incurred $3861 less in costs over 5 years.

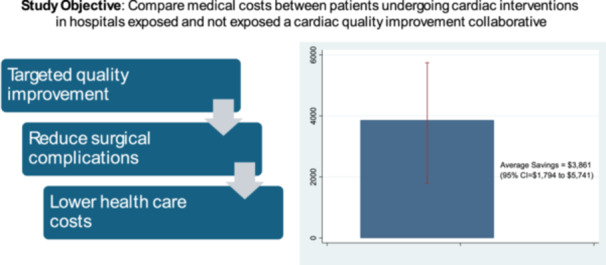

## 1 Introduction

The Cardiac Care Outcomes Assessment Program (COAP) is a data‐driven, physician‐led collaborative quality improvement program under the auspices of the Foundation for Health Care Quality, a nonprofit organization based in the Pacific Northwest that seeks to support hospitals and clinicians in achieving the highest levels of quality and patient safety. COAP is an approved Coordinated Quality Improvement Program under Washington State law, RCW 43.70.510 and focuses on care and outcomes in hospitals that perform percutaneous coronary intervention (PCI) and/or adult cardiac surgery. Since 2010, all hospitals that perform these procedures in Washington State have participated in Cardiac COAP, with the exception of the Veterans Affairs Medical Center in Seattle.

To achieve its mission, COAP collects comprehensive clinical and procedural data from participating hospitals to track quality indicators, which are publicly reported and targeted for regional quality improvement. As a key component of the collaborative process, COAP invites high‐performing providers to share best practices with all members at regular program meetings. These efforts that target improvement in the quality of cardiac care can reduce surgical complications, avert avoidable utilization such as hospital admissions, and improve long‐term patient outcomes. These improvements in quality can subsequently translate into lower costs to healthcare payers. While most evaluations have focused on how quality improvement collaboratives (QICs) such as COAP influence clinical outcomes, there is limited knowledge of the potential economic benefits. Another knowledge gap is whether the potential benefits of QICs are experienced by patients across different racial groups. This is significant given the increased focus of health systems to address structural and racial barriers to high quality care.

Previous studies have indicated public reporting on quality of care can stimulate quality improvement by promoting transparency and facilitating the informed choice of providers by patients [1]. By quantifying performance, low‐performing facilities are able to compare to benchmarks set by higher‐performing sites and set goals for practice change [2]. Radisic et al. point to the importance of process measures for quality improvement given these measures are sensitive to practice changes, are often easy to report and interpret, and are useful in understanding the nature of care [3]. In a systematic review of 27 studies, 14 found public reporting improved clinical outcomes [4]. This includes greater access to coronary artery bypass graft (CABG) surgery [5] reducing cardiac readmissions [6, 7], and predicted mortality among percutaneous coronary intervention (PCI) patients at negative outlier institutions [8]. However, to our knowledge, none of these prior studies examined the potential economic benefits of performance reporting.

To address this question, we sought to compare total medical costs between eligible patients undergoing cardiac interventions in hospitals in Washington State with similar patients undergoing intervention in other states. Empirical analyses examined total costs for 5 years following PCI or CABG. We apply an econometric approach that accounts for potential differences in costs that may arise from survival differences between groups. Notably, if Washington State patients survive markedly longer than comparable non‐Washington patients, then total healthcare costs may be higher due to improved survival, and not poorer care quality. To our knowledge, this is the first study to decompose survival and utilization effects in examining the potential economic benefits of QICs. Findings from this study will provide insights into the business case for QICs for cardiac care and inform the development of collaboratives for other care types.

## 2 Methods

The primary data source was Medicare fee‐for‐service (FFS) administrative and claims data accessed through the CMS Integrated Data Repository (IDR). The IDR is a high‐volume data warehouse that contains demographic and enrollment information on Medicare beneficiaries, all Medicare Parts A, B, C, D, and DME claims, and other ancillary sources. Inpatient and outpatient claims contain key data fields, including diagnosis and procedure codes, dates of service, and reimbursement amounts.

We examined all patients who underwent a PCI or CABG at any time over the period January 1, 2013, through September 30, 2020, using diagnosis codes from prior research [9]. Patients were also required to have been continuously enrolled in FFS Medicare in the 12 months before the intervention to ensure complete measurement of covariate data. We identified 2 533 112 patients (45 493 in Washington State and 2 490 213 in other states) meeting the inclusion criteria. In econometric analyses, we randomly selected 10% of these patients for analysis to increase computational feasibility.

Our econometric analysis requires jointly modeling patient‐quarter costs and mortality. The primary outcome was total medical costs in the 5 years following intervention (PCI or CABG) measured from the perspective of the public payer, which excludes any patient out‐of‐pocket expenditures (e.g., copayments, co‐insurance). Medical costs include all outpatient, inpatient and post‐acute care reimbursed by FFS Medicare. All costs were adjusted for inflation using the Consumer Price Index (CPI), which has been shown to be a more appropriate measure than the medical component of the CPI [10]. We examined total medical costs to comprehensively capture all potential follow‐up care related to cardiac intervention. This may include averted noncardiac care related to the initial procedure such as bleeding events, acute kidney injury, vascular injury, post‐procedural strokes, or other complications. To facilitate the econometric analysis, we partitioned costs into quarterly intervals starting from the day after surgical intervention through the earliest of the following: date of death, 5‐year follow‐up, or September 30, 2020, the end of our data capture. Mortality was defined as a binary variable denoting whether the patient's date of death fell within the time interval represented by a patient‐quarter observation.

The key exposure variable was a dichotomous variable indicating whether patients received cardiac intervention from a hospital located in Washington State. All non‐VA hospitals in Washington State participated in COAP. We defined exposure based on hospital location because our hypothesis is that components of the QIC impact the quality of CABG/PCI, which can reduce complications and avoidable utilization that can translate into economic benefit.

Our econometric analyses adjusted for several factors capturing patient demographics, comorbidity, and characteristics of patients' residence area in the 1 year before surgical intervention. Patient characteristics included age, gender, race/ethnicity, dual Medicaid enrollment, and the original reason for Medicare eligibility. Race was measured using data from the Medicare Enrollment database, which is populated using information from the Social Security Administration [11]. In addition, models adjusted for utilization in the previous year, including number of outpatient visits and hospitalization. Patient comorbidity was measured using 20 indicators from the validated Gagne comorbidity index [12].

Characteristics of patients' residence areas were derived by linking patients' residence ZIP codes with county Federal Information Processing Standard codes. Specific county‐level variables included the percentage of adults below the poverty line in the prior 12 months, median household income, the percentage of age 25+ adults with some college or an associate's degree, the percentage of the population that does not speak English, and whether the area was classified as rural.

To compare differences in downstream costs 5 years after surgical intervention, we applied a multipart estimator developed in prior econometric research [13]. Notably, this estimator accounts for the fact that the exact timing of death is stochastic and has a marked impact on downstream costs since costs are not accumulated after the date of death. To address this phenomenon, this multipart estimator seeks to compare expected costs between groups over the full 5‐year period and accounts for mortality risk by weighting costs by the probability of surviving to a future time period. The estimator also addresses elevated costs before death.

To operationalize this estimator, we used a parametric survival model to estimate patients' probability of surviving to a future quarter (i.e., the survival function), and the probability of dying in a given quarter (i.e., the hazard function). Next, we modeled expected costs in a given quarter among patient‐quarter observations where death was observed, using a generalized linear model (GLM) adjusting for covariates and a continuous variable denoting the proportion of the quarter a patient survived. Finally, we modeled expected cost in a given quarter among patient‐quarter observations where a full quarter of cost was observed, also using a GLM and adjusting for covariates.

We then calculated expected costs starting from the first quarter following cardiac intervention through Year 5. In each quarter, expected costs were calculated as the weighted average of adjusted costs conditional on survival and death, respectively. Adjusted costs in the event of survival were calculated as the predicted value from the third part of the multipart model. Similarly, adjusted costs in the event of death are calculated as the predicted value from the second part of the multipart model. Costs in the event of survival are weighted by the predicted probability of surviving the full quarter, as estimated by the parametric survival model. Similarly, costs in the event of death are weighted by the probability of dying within the quarter, also derived from the parametric survival model.

To derive total 5‐year costs, we summed expected quarterly costs, weighting each quarterly estimate by the probability of surviving to each quarter, produced from the parametric survival model. The difference in 5‐year medical expenditure between respective groups of patients receiving intervention within and outside of Washington State was calculated by conducting the post‐estimation procedure described above, conditional on the two levels of the treatment variable. Standard errors for treatment effect estimates were calculated using a bootstrap procedure [14]. All statistical analyses were performed using SAS Enterprise Guide Version 8.3 (SAS Institute Inc., Cary, NC). A nominal *p*‐value of 0.05 was used to assess all statistical hypotheses.

Geographic variation in Medicare reimbursement exists in part due to differences in delivering health services across regions. To assess the extent to which potential differences in costs between groups are driven by differences in Medicare payment rates, we analyzed publicly available data on Medicare costs at the state‐level [15]. For each state we took the ratio of total observed costs to total standardized costs. The latter excludes geographical differences in labor costs and practice expenses. A ratio of > 1 indicates state‐level payment rates, on average, were greater than standardized Medicare payment rates. We then took a weighted average of state‐level ratios with the proportion of all Medicare beneficiaries in each state serving as weights. We assessed whether the ratio for Washington State was greater than the national average, which would reflect higher Medicare payment rates in Washington State compared to the rest of the United States.

The distribution of quarter costs exhibited a high degree of skewness. Thus, in secondary analysis, we assess whether potential cost differences were driven by high‐cost outliers. This was accomplished by repeating all analyses removing the highest 1% and 5% of observations in each group.

## 3 Results

Of the 2.5 million patients in our study sample, 44 376 (1.75%) received cardiac intervention in Washington State (Table 1). Overall, patients were 72.5 years of age (standard deviation [SD] = 9.0), 64.6% were male and 86.4% were White race. The three most common comorbidities were hypertension (20.5%), uncomplicated diabetes (8.76%), and fluid/electrolyte disorder (5.86%). Characteristics were largely similar between WA and non‐WA patients with race being the only variable where meaningful differences were observed (defined as a standardized mean difference [SMD] > 0.1). Specifically, WA patients were less likely to be Black (1.87% vs. 7.18%, SMD = −0.21) and more likely to be White (89.9% vs. 86.36%, SMD = 0.10)

**Table 1:** Descriptive statistics among Medicare beneficiaries in Washington and non‐Washington states.

	All (*n* = 2 533 112)	WA (*n* = 44 376)	Non‐WA (*n* = 2 488 736)	SMD
Age (mean/SD)	72.50 (9.00)	73.06 (8.69)	72.49 (9.01)	0.06
Male (%)	64.60	67.98	64.54	0.07
*Race*				
White (%)	86.42	89.90	86.36	0.10
Black (%)	7.08	1.87	7.18	−0.21
Other (%)	6.50	8.23	6.46	0.07
Dual eligibility with Medicaid (%)	18.06	16.04	18.10	−0.05
*Reason for medicare eligibility*				
Aged without ESRD (%)	85.39	87.54	85.35	−0.01
Aged with ESRD (%)	2.29	2.15	2.29	0.06
Disabled without ESRD (%)	10.26	8.47	10.29	−0.06
Disabled with ESRD (%)	1.48	1.23	1.49	−0.02
ESRD only (%)	0.48	0.50	0.48	0.00
*Comorbidity flags*				
AIDS (%)	0.03	0.03	0.03	0.00
Alcohol abuse (%)	0.44	0.48	0.44	0.01
Deficiency anemias (%)	3.13	2.44	3.15	−0.04
Rheumatoid arthritis (%)	0.71	0.84	0.71	0.02
Chronic blood loss anemia (%)	0.15	0.16	0.15	0.00
Congestive heart failure (%)	0.91	0.79	0.92	−0.01
Chronic pulmonary disease (%)	5.63	5.09	5.64	−0.02
Coagulopathy (%)	2.28	2.13	2.28	−0.01
Depression (%)	1.59	1.52	1.59	−0.01
Diabetes without chronic complications (%)	8.76	7.50	8.79	−0.05
Diabetes with chronic complications (%)	1.86	1.91	1.86	0.00
Drug abuse (%)	0.25	0.42	0.25	0.03
Hypertension (%)	20.51	19.25	20.53	−0.03
Hypothyroidism (%)	3.05	2.93	3.06	−0.01
Liver disease (%)	0.34	0.33	0.35	0.00
Lymphoma (%)	0.18	0.19	0.18	0.00
Fluid and electrolyte disorders (%)	5.86	5.56	5.86	−0.01
Metastatic cancer (%)	0.14	0.11	0.14	−0.01
Other neurological disorders (%)	1.26	1.27	1.26	0.00
Obesity (%)	3.74	3.78	3.74	0.00
Paralysis (%)	0.40	0.39	0.40	0.00
Peripheral vascular disease (%)	3.41	3.22	3.41	−0.01
Psychoses (%)	0.54	0.51	0.54	0.00
Pulmonary circulation disease (%)	0.14	0.13	0.14	0.00
Renal failure (%)	5.21	4.90	5.22	−0.01
Solid tumor without metastasis (%)	0.39	0.35	0.39	−0.01
Peptic ulcer disease (%)	0.00	0.00	0.00	0.00
Valvular disease (%)	0.25	0.26	0.25	0.00
Weight loss (%)	0.73	0.40	0.74	−0.04
*County‐level measures*				
Adults below poverty line in prior 12 months (mean/SD)	14.39 (8.23)	11.49 (4.10)	14.46 (8.26)	−0.81
Median household income (thousands) (mean/SD)	62.52 (14.46)	72.07 (10.25)	62.30 (14.48)	0.66
Adults 25+ with associate's degree or above (mean/SD)	60.39 (10.74)	68.68 (9.37)	60.20 (10.69)	0.89
Percent of population not speaking English (mean/SD)	1.44 (7.46)	0.56 (1.78)	1.46 (7.50)	−0.70
Residing in noncore county (%)	5.78	2.14	5.86	−0.19

Abbreviations: ESRD = end stage renal disease, SMD = standardized mean difference, WA = Washington.

Mean total costs declined in the quarters following cardiac intervention (Table 2 and Supporting Information S1: Appendix Figure 1). Among all patients, mean quarterly costs were $9783 (SD = $20 117). Mean costs declined to $2975 (SD = $10 069) among the 2.1 million patients still alive at Quarter 11 and to $1565 (SD = $7392) among the 1.8 million patients still alive at Quarter 20. In each follow‐up quarter, costs were lower among Washington State, compared to non‐Washington State patients. In Quarter 1, mean costs were $9676 (SD = $20 446) among Washington State patients compared to $9784 (SD = $20 111) among non‐Washington State patients. This difference converged slightly over time where mean costs among Washington State patients were $1436 (SD = $6837) compared to $1568 (SD = $7401) for non‐Washington State patients.

**Table 2:** Unadjusted medical expenditures by follow‐up year.

	All			WA			Non‐WA		
	Mean	SD	*N*	Mean	SD	*N*	Mean	SD	*N*
Quarter 1	$9783	$20 117	2 533 112	$9676	$20 446	44 376	$9784	$20 111	2 488 736
Quarter 6	$3919	$11 569	2 222 824	$3591	$11 203	38 858	$3925	$11 576	2 183 966
Quarter 11	$2975	$10 069	2 066 663	$2707	$9389	36 143	$2980	$10 081	2 030 520
Quarter 16	$2181	$8589	1 932 364	$1930	$7574	33 729	$2186	$8606	1 898 635
Quarter 20	$1565	$7392	1 842 099	$1436	$6837	32 170	$1568	$7401	1 809 929

*Note:* All estimates are inflation‐adjusted to represent 2020 constant dollars.

**Figure 1:**
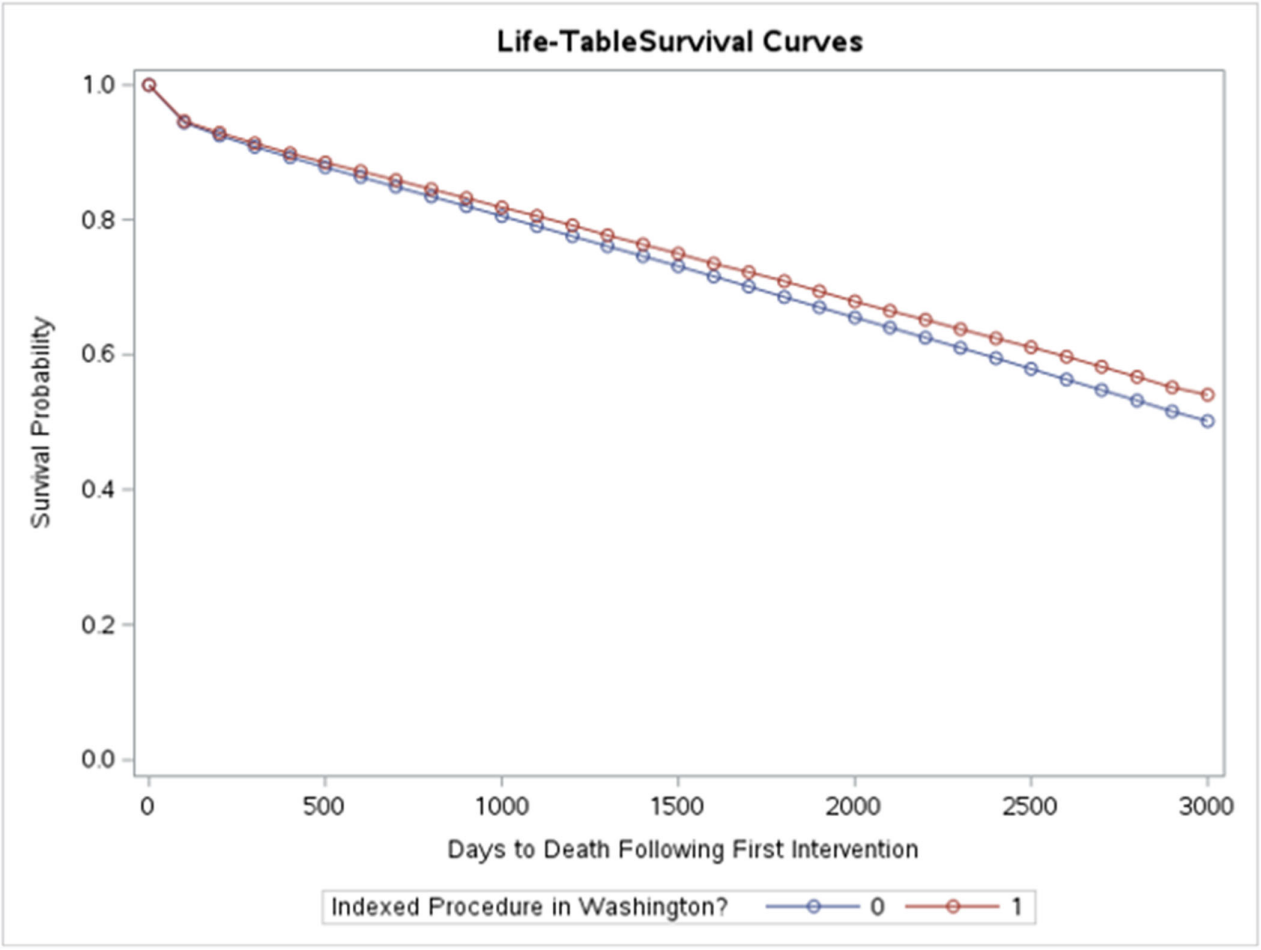
Kaplan−Meier curve summarizing time to death by group.

Kaplan−Meier curves indicate unadjusted rates of survival were greater among patients in Washington State (Figure 1). By 1800 days post‐intervention, the unadjusted probabilities of survival were 0.706 and 0.685 among Washington and non‐Washington patients, respectively.

In adjusted analysis applying the multipart model, we found expected total medical costs in the 5 years following cardiac intervention were $3861 lower (95% confidence interval [CI] = $1794 to $5741] among Washington State patients compared to patients in other states (Table 3). Lower downstream costs for Washington State patients were largely driven by lower costs in quarters where death was not observed (Table 4). Specifically, in the GLM model for costs, the coefficient for Washington State was negative and statistically significant (−0.0568, *p* < 0.001). In contrast, we found costs in quarters where death was observed, and the likelihood of death in a given quarter were not different between Washington and non‐Washington State patients.

**Table 3:** Estimated total adjusted 5‐year cost differences.

	Average COAP savings	95% confidence interval	
		Lower	Upper
All patients	$3861	$1794	$5741
Excluding top 1% of cost observations	$4249	$2234	$6264
Excluding top 5% of cost observations	$3405	$2268	$4950

**Table 4:** Parameter estimates for WA versus non‐WA from components of multipart model.

Model	Estimate	SE	Lower 95%	Upper 95%
Median survival years	−0.0494	0.0272	−0.1025	0.0038
Cost in death quarters	0.0111	0.0364	−0.0604	0.0825
Cost in non‐death quarters	−0.0568	0.0050	−0.0665	−0.0471

*Note:* Estimates inflation‐adjusted to represent 2020 constant dollars.

In sensitivity analysis, the weighted average ratio of total Medicare costs to total standardized Medicare costs was 1.04 compared to 1.08 in Washington State. This indicates prices for health services were higher in Washington State compared to the national average.

Patients undergoing cardiac intervention continued to incur lower total 5‐year costs after removing the top 1% and 5% of cost observations in each of the groups.

## 4 Discussion

This economic evaluation examined the 5‐year downstream costs following cardiac intervention (PCI or CABG) for coronary artery disease (CAD). To estimate the potential economic benefits and cost savings of the Cardiac COAP collaborative, we compared cost outcomes between respective groups of patients receiving cardiac intervention in Washington State and other states. We found patients in Washington State had lower expected costs following the intervention. The $3861 cost reduction identified in our analysis, if extrapolated over the approximately 2.5 million patients undergoing PCI or CABG nationally translates to a $9.7 billion reduction in costs to the Medicare program. This represents about 1.1% of the $829.5 billion in total Medicare spending in 2020.

Lower downstream costs among patients receiving intervention in Washington State are at least in part attributable to COAP for several reasons. First, during the study period, all non‐VA hospitals in Washington State participated in the COAP program. COAP has been associated with improved cardiac quality through markedly fewer blood transfusions in patients undergoing cardiac surgery, a reduction in postoperative ventilation time, and increased radial arterial access in patients undergoing PCI as recent examples [16, 17]. In addition, prior research has found that COAP is associated with reductions in inappropriate PCIs [18], year‐over‐year improvement in CABG mortality among poorer‐performing facilities, and an increasing trend in the proportion of CABG patients extubated within 6 h [19]. A study found patients undergoing CABG at hospitals participating in an informational collaborative targeting pneumonia prevention practices experienced lower pneumonia infection rates [20]. In sensitivity analysis, we found that lower costs among Washington State patients were not due to lower Medicare payment rates for health services. In fact, on average, the ratio of total to standardized cost in Washington State was higher compared to other states. This suggests the effects of COAP and other state‐level effects more than offset higher Medicare payment rates for services delivered in Washington State.

This study contributes to the substantial evidence gap on the economic impacts of cardiology QICs. Findings from this study coincide with a limited literature that has examined potential downstream cost savings associated with QICs. For example, Wicke et al. found a collaborative care program to improve coordination between general practitioners and cardiologists was associated with a reduction in cardiology disease‐specific costs over 1‐year of follow‐up [21]. Outside of cardiology, a systematic review [22] identified two cost analyses that showed reductions in costs attributable to QICs for Parkinson's disease [23] and neonatal intensive care [24]. Three additional studies identified QICs for diabetes, obstetric and newborn care, and long‐term care met the threshold for cost‐effectiveness.

This study has two notable limitations. First, our econometric models controlled for a wide range of patient characteristics that seek to increase the comparability of patients receiving intervention within and outside of Washington State, respectively. It is possible that cost differences between groups may capture the influence of other unobserved factors. Second, our analyses specifically examined economic impacts among the population of patients enrolled in FFS Medicare. It is unknown to what degree cost differences between groups translate to other populations. Measuring differences in downstream costs among patients enrolled in Medicare Advantage, commercial insurance, and other payers represents an area of future research.

In summary, 5‐year total healthcare costs following cardiac intervention for CAD were lower among patients in Washington State, compared to other states. Lower costs were not due to geographical variations in the price of health services nationally. These cost differences are likely due, in part, to Cardiac COAP, given the comprehensive participation by all non‐VA hospitals in Washington State and its long history of quality improvement efforts. Future research should examine whether comparable cost differences exist in other populations with a high burden of CAD.

## Conflicts of Interest

The authors declare no conflicts of interest.

## Supporting information

Supporting information.

## Data Availability

Research data are not shared.

## References

[1] M. Cacace , M. Geraedts , and E. Berger , “Public Reporting as a Quality Strategy,” in Improving Healthcare Quality in Europe: Characteristics, Effectiveness and Implementation of Different Strategies, eds. R. Busse , N. Klazinga , D. Panteli , and W. Quentin (Copenhagen, Denmark: World Health Organization, Regional Office for Europe, 2019). 31721544

[2] P. Shekelle , “Performance Measurement for Health System Improvement.” in Performance Measurement for Health System Improvement, eds. P. Smith , E. Mossialos , I. Papanicolas , and S. Leatherman (Cambridge, UK: Cambridge University Press, 2009).

[3] G. Radisic , L. de la Perrelle , and K. Laver , “Methods of Capturing Process Outcomes in Quality Improvement Trials: A Systematic Review,” Journal for Healthcare Quality 44, no. 3 (2022): 131–151. 35119423 10.1097/JHQ.0000000000000336

[4] P. Campanella , V. Vukovic , P. Parente , A. Sulejmani , W. Ricciardi , and M. L. Specchia , “The Impact of Public Reporting on Clinical Outcomes: A Systematic Review and Meta‐Analysis,” BMC Health Services Research 16 (2016): 296. 27448999 10.1186/s12913-016-1543-y PMC4957420

[5] Z. Li , D. M. Carlisle , J. P. Marcin , et al., “Impact of Public Reporting on Access to Coronary Artery Bypass Surgery: The California Outcomes Reporting Program,” Annals of Thoracic Surgery 89, no. 4 (2010): 1131–1138. 20338320 10.1016/j.athoracsur.2009.12.073

[6] R. M. Werner and E. T. Bradlow , “Public Reporting on Hospital Process Improvements Is Linked to Better Patient Outcomes,” Health Affairs 29, no. 7 (2010): 1319–1324. 20606180 10.1377/hlthaff.2008.0770

[7] D. Dranove , D. Kessler , M. McClellan , and M. Satterthwaite , “Is More Information Better? The Effects of ‘Report Cards’ on Health Care Providers,” Journal of Political Economy 111, no. 3 (2003): 555–588.

[8] J. M. McCabe , K. E. Joynt , F. G. P. Welt , and F. S. Resnic , “Impact of Public Reporting and Outlier Status Identification on Percutaneous Coronary Intervention Case Selection in Massachusetts,” JACC: Cardiovascular Interventions 6, no. 6 (2013): 625–630. 23787236 10.1016/j.jcin.2013.01.140 PMC6948720

[9] A. L. Beatty , M. Truong , D. W. Schopfer , H. Shen , J. M. Bachmann , and M. A. Whooley , “Geographic Variation in Cardiac Rehabilitation Participation in Medicare and Veterans Affairs Populations: Opportunity for Improvement,” Circulation 137, no. 18 (2018): 1899–1908. 29305529 10.1161/CIRCULATIONAHA.117.029471 PMC5930133

[10] E. R. Berndt , D. M. Cutler , R. G. Frank , et al., “Price Indexes for Medical Care Goods and Services—An Overview of Measurement Issues,” in Medical Care Output and Productivity, eds. D. M. Cutler and E. R. Berndt (Chicago, IL: University of Chicago Press, 2001).

[11] O. F. Jarrín , A. N. Nyandege , I. B. Grafova , X. Dong , and H. Lin , “Validity of Race and Ethnicity Codes in Medicare Administrative Data Compared With Gold‐Standard Self‐Reported Race Collected During Routine Home Health Care Visits,” Medical Care 58, no. 1 (2020): e1–e8. 31688554 10.1097/MLR.0000000000001216 PMC6904433

[12] “Risk Adjustment,” Centers for Medicare and Medicaid Services, 2019, https://www.cms.gov/Medicare/Health-Plans/MedicareAdvtgSpecRateStats/Risk-Adjustors.html.

[13] A. Basu and W. G. Manning , “Estimating Lifetime or Episode‐of‐Illness Costs Under Censoring,” Health Economics 19, no. 9 (2010): 1010–1028. 20665908 10.1002/hec.1640

[14] B. E. Dowd , W. H. Greene , and E. C. Norton , “Computation of Standard Errors,” Health Services Research 49, no. 2 (2014): 731–750. 24800304 10.1111/1475-6773.12122 PMC3976195

[15] “Medicare Geographic Variation—by National, State & County,” Centers for Medicare and Medicaid Services, 2022, https://data.cms.gov/summary-statistics-on-use-and-payments/medicare-geographic-comparisons/medicare-geographic-variation-by-national-state-county/data.

[16] J. Brevig , J. McDonald , E. S. Zelinka , T. Gallagher , R. Jin , and G. L. Grunkemeier , “Blood Transfusion Reduction in Cardiac Surgery: Multidisciplinary Approach at a Community Hospital,” Annals of Thoracic Surgery 87, no. 2 (2009): 532–539. 19161774 10.1016/j.athoracsur.2008.10.044

[17] A. Kataruka , J. A. Doll , and R. S. Hira , “Public Reporting for Cardiac Procedures,” Journal of the American College of Cardiology 74, no. 17 (2019): 2218. 31648716 10.1016/j.jacc.2019.07.086

[18] S. M. Bradley , C. M. Bohn , D. J. Malenka , et al., “Temporal Trends in Percutaneous Coronary Intervention Appropriateness: Insights From the Clinical Outcomes Assessment Program,” Circulation 132, no. 1 (2015): 20–26. 26022910 10.1161/CIRCULATIONAHA.114.015156

[19] J. R. Goss , C. Maynard , G. S. Aldea , et al., “Effects of a Statewide Physician‐Led Quality‐Improvement Program on the Quality of Cardiac Care,” American Heart Journal 151, no. 5 (2006): 1033–1042. 16644333 10.1016/j.ahj.2005.06.035

[20] D. S. Likosky , S. D. Harrington , L. Cabrera , et al., “Collaborative Quality Improvement Reduces Postoperative Pneumonia After Isolated Coronary Artery Bypass Grafting Surgery,” Circulation: Cardiovascular Quality and Outcomes 11, no. 11 (2018): e004756. 30571334 10.1161/CIRCOUTCOMES.118.004756 PMC6310019

[21] F. S. Wicke , B. Ditscheid , T. Breitkreuz , et al., “Clinical and Economic Outcomes of a Collaborative Cardiology Care Program,” American Journal of Managed Care 27, no. 4 (2021): e114–e122. 33877778 10.37765/ajmc.2021.88620

[22] L. de la Perrelle , G. Radisic , M. Cations , B. Kaambwa , G. Barbery , and K. Laver , “Costs and Economic Evaluations of Quality Improvement Collaboratives in Healthcare: A Systematic Review,” BMC Health Services Research 20, no. 1 (2020): 155. 32122378 10.1186/s12913-020-4981-5 PMC7053095

[23] B. R. Bloem , L. Rompen , N. M. Vries , A. Klink , M. Munneke , and P. Jeurissen , “ParkinsonNet: A Low‐Cost Health Care Innovation With a Systems Approach From The Netherlands,” Health Affairs 36, no. 11 (2017): 1987–1996. 29137501 10.1377/hlthaff.2017.0832

[24] J. A. Rogowski , J. D. Horbar , P. E. Plsek , et al., “Economic Implications of Neonatal Intensive Care Unit Collaborative Quality Improvement,” Pediatrics 107, no. 1 (2001): 23–29. 11134429 10.1542/peds.107.1.23

