# Supplementary material for: Association Between State‐Wide Cardiac Quality Improvement Program and Costs Following Intervention for Coronary Artery Disease
Source: Clin Cardiol. 2024 Nov 18;47(11):e70030. doi: 10.1002/clc.70030 (PMC11573734; doi:10.1002/clc.70030)

**Supplemental Appendix**

**Association of a State-Wide Cardiac Quality Improvement Program With Costs Following Cardiac Intervention for Coronary Artery Disease**

**Description of Econometric Approach to Examine Differences in 5-year Costs**

To estimate total adjusted five-year downstream costs and costs differences between patients receiving PCI/CABG in Washington versus non-Washington State hospitals, we applied the multi-part estimator developed by Basu and Manning (2010), *Health Economics*. This estimator separates cost differences between groups due to respective survival and utilization effects.

The estimator requires implementing three steps:

1. Fit a parametric survival model to calculate the probability of surviving to quarter *j*, denoted $\hat{S}_{j}\left( X \right)$, and the probability of dying between quarter *j* and *j+1*, denoted, $\hat{h}_{j}\left( X \right)$. The vector *X* includes exposure group (WA=0,1) and other patient covariates.
2. Model expected quarterly costs among patient-quarter observations where death was observed, denoted $\hat{\mu}_{1j}\left( X \right)$, using a generalized linear model (GLM).
3. Model expected quarterly costs among patient-quarter observations where costs over the full quarter were observed, denoted $\hat{\mu}_{2j}\left( X \right)$, also using a GLM.

To calculate expected total costs in quarter *j*, we calculated the weighted average of adjusted cost conditional on a) surviving the full quarter [$\hat{\mu}_{2j}\left( X \right)$], and b) dying within the quarter [$\hat{\mu}_{1j}\left( X \right)$], with adjusted hazard rates $\hat{h}_{j}\left( X \right)$ serving as weights. For each patient, expected costs in the 60 quarters of follow-up were summed together to generate total five-year expected costs. Specifically, we calculated the following for each patient using the method of recycled predictions:

$$\text{Total 5-year Cost}=\sum_{j=1}^{60} \hat{S}_{j}\left( X \right)[\hat{h}_{j}\left( X \right)*\hat{\mu}_{1j}\left( X \right)+\left( 1-\hat{h}_{j}\left( X \right) \right)*\hat{\mu}_{2j}\left( X \right)]$$

We then calculated expected 5-year costs attributable to hospitals’ exposure to the quality improvement collaborative as the difference in costs between patients receiving cardiac intervention in Washington State relative to patients receiving intervention outside of Washington State. This difference was a function of recycled predictions used calculate total expected costs [$\hat{\mu}_{1j}\left( X \right)$, $\hat{\mu}_{2j}\left( X \right)$, $\hat{S}_{j}\left( X \right)$ and $\hat{H}_{j}\left( X \right)$], and the difference in each of these quantities conditional on receiving cardiac intervention within and outside of Washington, respectively. Specifically, we calculated the following for each patient:

$$\frac{d\text{Total 5-year Cost}}{dWA}=\sum_{j=1}^{60} \frac{{d\hat{S}}_{j}\left( X \right)}{dWA}[\hat{h}_{j}\left( X \right)*\hat{\mu}_{1j}\left( X \right)+\left( 1-\hat{h}_{j}\left( X \right) \right)*\hat{\mu}_{2j}\left( X \right)]+\hat{S}_{j}\left( X \right)\left[ \frac{{d\hat{h}}_{j}\left( X \right)}{dWA}*(\hat{\mu}_{1j}\left( X \right)-\hat{\mu}_{2j}\left( X \right))+ \hat{h}_{j}\left( X \right)*\frac{{d\hat{\mu}}_{1j}\left( X \right)}{dWA}+(1-\hat{h}_{j}\left( X \right))*\frac{{d\hat{\mu}}_{2j}\left( X \right)}{dWA} \right]$$

We report this 5-year cost difference in Table 3 of the manuscript.

**Figure 1**: Mean inflation adjusted costs by group and quarter post-procedure.


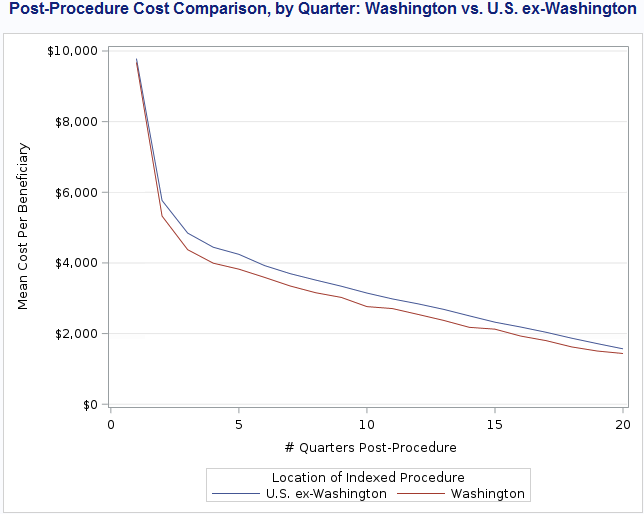

Supplement: Supplementary file 1 — Supporting information. [file CLC-47-e70030-s001.docx]
